# Temporal Trends in Inadequate Vegetable and Fruit Consumption Among Adolescents Aged 12–15 Years From 31 Countries in Asia, Africa, and the Americas

**DOI:** 10.1002/hsr2.70711

**Published:** 2025-04-21

**Authors:** Lee Smith, Guillermo F. López Sánchez, Mark A. Tully, Yvonne Barnett, Laurie Butler, Helen Keyes, Louis Jacob, Karel Kostev, Hans Oh, Masoud Rahmati, Jae Il Shin, Ai Koyanagi

**Affiliations:** ^1^ Centre for Health Performance and Wellbeing Anglia Ruskin University Cambridge UK; ^2^ Department of Public Health Sciences, Division of Preventive Medicine and Public Health, School of Medicine University of Murcia Murcia Spain; ^3^ School of Medicine Ulster University Londonderry Northern Ireland UK; ^4^ School of Psychology, Sport and Sensory Sciences Anglia Ruskin University Cambridge UK; ^5^ Research and Development Unit Parc Sanitari Sant Joan de Déu, Dr. Antoni Pujadas, Sant Boi de Llobregat Barcelona Spain; ^6^ AP‐HP, Université Paris Cité, Lariboisière‐Fernand Widal Hospital, Department of Physical Medicine and Rehabilitation Paris France; ^7^ Université Paris Cité, Inserm U1153, Epidemiology of Ageing and Neurodegenerative Diseases (EpiAgeing) Paris France; ^8^ University Clinic of Marburg Marburg Germany; ^9^ Suzanne Dworak Peck School of Social Work University of Southern California Los Angeles California USA; ^10^ Department of Physical Education and Sport Sciences, Faculty of Literature and Human Sciences Lorestan University Khoramabad Iran; ^11^ Department of Physical Education and Sport Sciences, Faculty of Literature and Humanities Vali‐E‐Asr University of Rafsanjan Rafsanjan Iran; ^12^ Department of Pediatrics Yonsei University College of Medicine Seoul South Korea; ^13^ Severance Underwood Meta‐Research Center, Institute of Convergence Science Yonsei University Seoul Republic of Korea

**Keywords:** adolescents, epidemiology, fruit and vegetable consumption, multicountry, temporal trends

## Abstract

**Background and Aims:**

A low intake of fruit and vegetable consumption has been found to be associated with a plethora of negative health outcomes in adolescents. However, there is a scarcity of literature on long‐term trends in fruit and vegetable intake in the adolescent population. Therefore, we examined this trend in a nationally representative sample of adolescents (12–15 years) attending school in 31 countries, including Africa, Asia, and the Americas, where investigation of such trends has been scarce.

**Methods:**

The present study analyzed data from the Global School‐based Student Health Survey 2003–2017. The prevalence (95% CI) of inadequate fruit and vegetable intake (i.e., consumption < 5 times/day) was calculated for each survey, and crude linear trends were examined by linear regression models for each country.

**Results:**

We analyzed data from students (*n* = 193,388) aged 12–15 years [mean (SD) age 13.7 (1.0) years; 49.0% boys]. A high overall prevalence of inadequate fruit and vegetable consumption was found (75%). We observed increasing, decreasing, and stable trends in 6, 3, and 22 countries, respectively. In countries where decreasing trends were found, this decrease was minimal. Moreover, the majority of countries with stable trends exhibited a high prevalence of inadequate fruit and vegetable intake across multiple years.

**Conclusion:**

Our data show that inadequate fruit and vegetable consumption among adolescents is a major global problem with almost no improvements being observed in recent years. Intensification of global efforts to combat inadequate fruit and vegetable consumption is necessary.

## 1 Introduction

Adequate fruit and vegetable consumption (defined as at least 400 g/day, i.e., five portions/day) [1] is associated with a plethora of positive health outcomes among adolescents, including, for example, protection against anxiety and depressive symptoms [2], reduction in inflammation and oxidative stress [3], obesity [4, 5], and metabolic syndrome [6, 7]. Importantly, adequate fruit and vegetable consumption during adolescence also provides protection against health complications in adulthood. For example, literature has shown that fruit and vegetable intake in adolescence is protective against breast cancer [3, 8], depression [9], diabetes [10, 11, 12, 13], and improves self‐rated health [3, 14] in adulthood.

Therefore, it is essential to understand the prevalence and temporal trends of fruit and vegetable consumption among adolescents for service planning and policy development in terms of improvements in diets. However, despite the well‐established health benefits of consuming fruit and vegetables during adolescence, there is little literature on its temporal trends. For example, one study used data from 488,951 adolescents aged 11–15 years from 33 mainly European and North American countries/regions between 2002 and 2010, and found an increase in daily fruit and vegetable consumption between 2002 and 2010 in the majority of countries [15]. Furthermore, another study used two cross‐sectional samples consisting of Western Australian secondary school students aged 12–17 years surveyed in 2009–2010 (*n* =  1501) and 2012–2013 (*n* =  1406) and found that there was almost no change in the percentage of adolescents who consumed an adequate amount of vegetables or fruits between the two waves [16]. Other studies have also examined trends in fruit and vegetable consumption among adolescents but exclusively in single Western high‐income countries (i.e., Norway, USA) [17, 18]. The main limitation of the previous studies is that the data are from Western high‐income countries (with some data from Western middle‐income countries), and there are no data from other settings. This is a major omission as improvement in diets, including fruit and vegetable consumption, is an important strategy to prevent future chronic diseases, which is increasing drastically, especially in low‐ and middle‐income countries (LMICs), and for which treatment options may be limited in this setting. Indeed, 77% of all non‐communicable deaths occur in LMICs [19]. Furthermore, compared to high‐income countries, LMICs may face challenges in affordability, accessibility, and safety of fruits and vegetables.

In light of the above rationale, the present study aimed to examine the temporal trend of fruit and vegetable consumption in a sample of 193,388 students aged 12–15 years from 31 countries in Africa, Asia, and the Americas (predominantly LMICs) where data on temporal trends of fruit and vegetable intake are scarce.

## 2 Methods

### 2.1 The Survey

The present study analyzed data from the Global School‐based Student Health Survey (GSHS). Survey details are available at https://www.who.int/teams/noncommunicable-diseases/surveillance/data and http://www.cdc.gov/gshs. In brief, the GSHS was designed by the WHO and the US Centers for Disease Control and Prevention (CDC), in collaboration with UN allies. The survey aimed to assess and quantify risk and protective factors in relation to predominant non‐communicable diseases. For the selection process in each participating country, a standardized two‐stage probability sampling design was employed. Firstly, schools were selected with probability proportional to size sampling. The next stage randomly selected classrooms, including students aged 13–15 years, within each selected school. However, all students in the selected classrooms were able to participate in the survey regardless of age. Data collection was carried out during one class period. In each country, the surveys were translated into the local language. Students reported their responses on computer scannable sheets with multiple‐choice response options. A national government administration and an institutional review board or ethics committee approved GSHS surveys in each respective country. The present analysis used only publicly available data, and thus, ethical approval for this analysis was not required. All surveys were anonymous, and participation was voluntary. Explicit informed consent was obtained as appropriate from the students, parents, and/or school officials. Data was weighted for nonresponse and probability selection [20, 21].

Data sets, including variables pertaining to analyses, were selected. Moreover, data sets needed to contain data on at least two waves from the same country. Ultimately, 31 countries were incorporated into this study. By country, the survey year, income level, response rate, and sample size are presented in Table 1. The surveys included in this study were carried out between 2003 and 2017 and predominantly LMICs.

**Table 1:** Survey characteristics.

Region	Country	Country income	Year	Response rate (%)	*N* a
AFR	Benin	L	2009	90	1170
		L	2016	78	717
	Mauritius	UM	2011	82	2074
		UM	2017	84	1955
	Seychelles	UM	2007	82	1154
		H	2015	82	2061
	Swaziland	LM	2003	96	6866
		LM	2013	97	1318
AMR	Anguilla	NA	2009	84	701
		NA	2016	88	564
	Argentina	UM	2007	77	1537
		UM	2012	71	21,528
	Guatemala	LM	2009	81	4495
		LM	2015	82	3611
	Guyana	LM	2004	80	1070
		LM	2010	76	1973
	Jamaica	UM	2010	72	1204
		UM	2017	60	1061
	Suriname	UM	2009	89	1046
		UM	2016	83	1453
	Trinidad & Tobago	H	2007	78	2450
		H	2011	90	2363
		H	2017	89	2763
	Uruguay	UM	2006	71	2882
		H	2012	77	2869
EMR	Egypt	LM	2006	87	4981
		LM	2011	85	2364
	Jordan	LM	2004	95	1848
		LM	2007	99.8	1648
	Kuwait	H	2011	85	2298
		H	2015	78	2034
	Lebanon	UM	2005	88	4524
		UM	2011	87	1982
		UM	2017	82	3347
	Morocco	LM	2006	84	1986
		LM	2010	92	2405
		LM	2016	91	3975
	Oman	UM	2005	97	2426
		H	2010	89	1000
		H	2015	92	1669
	UAE	H	2005	89	12,819
		H	2010	91	2302
		H	2016	80	3471
	Yemen	L	2008	82	905
		LM	2014	75	1553
SEAR	Indonesia	LM	2007	93	3022
		LM	2015	94	8806
	Maldives	LM	2009	80	1981
		UM	2014	60	1781
	Myanmar	L	2007	95	2227
		LM	2016	86	2237
	Sri Lanka	LM	2008	89	2504
		LM	2016	89	2254
	Thailand	LM	2008	93	2675
		UM	2015	89	4132
WPR	Cook Islands	NA	2011	84	849
		NA	2015	65	366
	Fiji	LM	2010	90	1495
		UM	2016	79	1537
	Philippines	LM	2003	84	4198
		LM	2007	81	3484
		LM	2011	82	3845
		LM	2015	79	6162
	Samoa	LM	2011	79	2200
		LM	2017	59	1058
	Tonga	LM	2010	80	1946
		UM	2017	90	2067
	Vanuatu	LM	2011	72	852
		LM	2016	57	1288

*Note:* Data on country income were not available from Cook Islands and Anguilla.

Abbreviations: AFR, African Region; AMR, Region of the Americas; EMR, Eastern Mediterranean Region; H, high income; L, low income; LM, lower middle income; SEAR, South‐East Asia Region; UM, upper middle income; WPR, Western Pacific Region.

^a^ Based on a sample aged 12–15 years.

### 2.2 Fruit and Vegetable Consumption

Fruit consumption was assessed by the question “During the past 30 days, how many times per day did you usually eat fruit?” and vegetable consumption by the question “During the past 30 days, how many times per day did you usually eat vegetables?” Both questions included country‐specific examples of fruits or vegetables as references. The recommended intake of fruit and vegetables based on the Joint FAO/WHO Expert Consultation on Diet, Nutrition, and the Prevention of Chronic Diseases is ≥ 5 servings of fruits/vegetables per day. However, given that the GSHS only provided information on the frequency of intake with no information on quantity, we were unable to create a variable that corresponds completely to the FAO/WHO recommendation. Rather, as in previous GSHS publications, we used ≥ 5 times/day of fruits/vegetables as a proxy of adequate intake of fruit and vegetables, while not reaching this amount of fruit/vegetable consumption was considered inadequate intake [22].

### 2.3 Statistical Analysis

Analyses were conducted using Stata 14.2 (Stata Corp LP, College Station, Texas). Students aged between 12 and 15 years were included, as the majority of students fell within this age range. Details on exact age outside this range were not reported. For each survey, the prevalence and 95% CI of inadequate fruit/vegetable intake were derived for the overall sample and by sex. Crude linear trends in inadequate fruit/vegetable intake were investigated by employing linear regression analyses across survey waves within the same country, and subsequently, regression coefficients (beta) and 95% CI for every 1‐year change were produced. Next, using the survey as a continuous variable *p* for trends was estimated. For interaction analyses, an interaction term (survey year X sex) was included in the model to ascertain whether trends varied between boys and girls. All analyses considered sampling weights and the clustered sampling design of the surveys. The sampling design was addressed in the analysis using Taylor's linearization method. We have employed similar analyses in previous published works [20, 21].

## 3 Results

**Figure 1:**
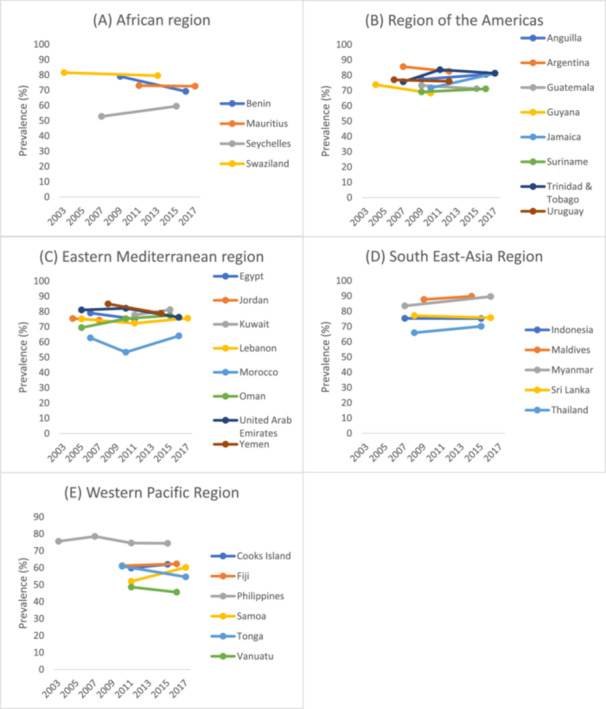
Trends in prevalence (%) of inadequate fruit/vegetable consumption by country and region.

Data were analyzed from a total of 193,388 students aged 12–15 years [mean (SD) age 13.7 (1.0) years]. A total of 49.0% of the sample were boys. The mean prevalence of inadequate fruit/vegetable consumption across all surveys was 75.0%. Overall sample prevalence trends, including boys and girls, of inadequate fruit/vegetable consumption are presented in Table 2 and Figure 1. The prevalence of inadequate fruit/vegetable consumption was highest in the Maldives in 2014 (89.8%) and was lowest in Vanuatu in 2016 (45.6%). Among all countries included in the analysis, we observed significant increasing trends of inadequate fruit/vegetable consumption in six countries and significant decreasing trends in three countries. We observed significant increasing trends in Seychelles, Jamaica, Trinidad & Tobago, Oman, Myanmar, and Samoa. We observed significant decreasing trends in Benin, the United Arab Emirates, and Tonga. In the other 22 countries included, no significant trends were identified. Prevalence trends of inadequate fruit/vegetable consumption by sex can be found in Table S1 of the Appendix. Similar trends by sex were observed in most countries. However, a significant interaction in trends by sex was seen in three countries. Specifically, a significant decreasing trend of inadequate fruit/vegetable consumption was found among boys in Guyana and Vanuatu, while an increasing trend was only observed among boys in Samoa.

**Table 2:** Trends in prevalence (%) of inadequate fruit/vegetable consumption in 31 countries (overall).

Country	Year	%	[95% CI]	Beta^a^	[95% CI]	*p* for trend^a^
AFR						
Benin	2009	79.0	[75.8, 82.0]	−1.40	[−2.28, −0.53]	0.003
	2016	69.2	[64.0, 74.0]			
Mauritius	2011	73.0	[69.4, 76.3]	−0.07	[−0.91, 0.77]	0.862
	2017	72.6	[69.1, 75.8]			
Seychelles	2007	52.8	[52.1, 53.4]	0.84	[0.40, 1.27]	< 0.001
	2015	59.5	[56.0, 62.8]			
Swaziland	2003	81.5	[79.5, 83.4]	−0.21	[−0.60, 0.19]	0.307
	2013	79.5	[75.8, 82.7]			
AMR						
Anguilla	2009	77.3	[77.3, 77.3]	0.45	[−0.05, 0.95]	0.077
	2016	80.5	[76.8, 83.6]			
Argentina	2007	85.6	[82.0, 88.6]	−0.63	[−1.35, 0.08]	0.083
	2012	82.5	[81.0, 83.8]			
Guatemala	2009	73.3	[70.9, 75.5]	−0.35	[−0.89, 0.20]	0.208
	2015	71.2	[68.9, 73.4]			
Guyana	2004	73.8	[69.0, 78.1]	−0.92	[−2.03, 0.19]	0.100
	2010	68.3	[63.7, 72.6]			
Jamaica	2010	71.7	[68.4, 74.8]	1.36	[0.61, 2.11]	0.001
	2017	81.2	[77.0, 84.8]			
Suriname	2009	69.1	[65.5, 72.4]	0.29	[−0.35, 0.92]	0.366
	2016	71.1	[68.5, 73.4]			
Trinidad & Tobago	2007	75.7	[73.3, 77.9]	0.52	[0.22, 0.81]	0.001
	2011	83.6	[80.9, 85.9]			
	2017	81.3	[79.3, 83.3]			
Uruguay	2006	77.0	[74.6, 79.2]	−0.17	[−0.68, 0.34]	0.519
	2012	76.0	[74.0, 77.8]			
EMR						
Egypt	2006	79.0	[74.6, 82.8]	−0.79	[−2.15, 0.56]	0.245
	2011	75.1	[69.5, 79.9]			
Jordan	2004	75.4	[71.9, 78.6]	−0.36	[−1.98, 1.27]	0.655
	2007	74.3	[70.9, 77.4]			
Kuwait	2011	77.7	[74.3, 80.7]	0.88	[−0.21, 1.97]	0.111
	2015	81.2	[78.3, 83.8]			
Lebanon	2005	75.1	[73.5, 76.6]	0.06	[−0.19, 0.30]	0.654
	2011	72.3	[69.5, 75.0]			
	2017	75.6	[73.1, 77.9]			
Morocco	2006	62.7	[60.4, 64.9]	0.36	[−0.09, 0.82]	0.115
	2010	53.3	[50.7, 55.9]			
	2016	64.0	[60.3, 67.7]			
Oman	2005	69.4	[66.5, 72.1]	0.83	[0.40, 1.27]	< 0.001
	2010	75.3	[71.1, 79.0]			
	2015	77.4	[74.0, 80.5]			
United Arab Emirates	2005	81.0	[80.0, 82.0]	−0.47	[−0.78, −0.17]	0.003
	2010	82.1	[79.5, 84.5]			
	2016	76.2	[73.0, 79.1]			
Yemen	2008	85.0	[78.8, 89.7]	−1.06	[−2.28, 0.15]	0.084
	2014	78.7	[73.9, 82.8]			
SEAR						
Indonesia	2007	75.3	[72.6, 77.8]	0.00	[−0.41, 0.40]	0.983
	2015	75.2	[73.3, 77.1]			
Maldives	2009	87.7	[84.3, 90.4]	0.42	[−0.28, 1.13]	0.240
	2014	89.8	[87.9, 91.4]			
Myanmar	2007	83.5	[80.1, 86.4]	0.68	[0.25, 1.10]	0.002
	2016	89.6	[87.4, 91.5]			
Sri Lanka	2008	77.1	[74.2, 79.9]	−0.18	[−0.71, 0.34]	0.489
	2016	75.7	[72.7, 78.5]			
Thailand	2008	65.9	[61.8, 69.8]	0.59	[−0.14, 1.33]	0.112
	2015	70.1	[67.0, 73.0]			
WPR						
Cooks Island	2011	59.8	[59.8, 59.8]	0.54	[−0.70, 1.78]	0.381
	2015	62.0	[57.0, 66.6]			
Fiji	2010	61.2	[56.9, 65.4]	0.18	[−1.01, 1.36]	0.764
	2016	62.3	[56.9, 67.4]			
Philippines	2003	75.6	[72.9, 78.0]	−0.22	[−0.48, 0.04]	0.104
	2007	78.5	[76.8, 80.2]			
	2011	74.6	[71.5, 77.4]			
	2015	74.4	[72.2, 76.4]			
Samoa	2011	52.0	[48.9, 55.2]	1.36	[0.63, 2.10]	< 0.001
	2017	60.2	[57.2, 63.1]			
Tonga	2010	60.9	[57.9, 63.9]	−0.91	[−1.46, −0.36]	0.001
	2017	54.6	[52.2, 57.0]			
Vanuatu	2011	48.6	[43.8, 53.4]	−0.59	[−1.86, 0.68]	0.357
	2016	45.6	[41.7, 49.6]			

Abbreviations: AFR, African Region; AMR, Region of the Americas; CI, confidence interval; EMR, Eastern Mediterranean Region; SEAR, South‐East Asia Region; WPR, Western Pacific Region.

^a^ The beta and *p* for trend are based on linear regression, including survey year as a continuous variable. The beta can be interpreted as the average percentage point change in prevalence per year.

## 4 Discussion

### 4.1 Main Findings

In the present study, including 193,388 students aged 12–15 years across 31 countries, which were mainly LMICs, the prevalence of inadequate fruit and vegetable intake was very high (75%). It is important to note here that our measure of inadequate fruit and vegetable intake was based on times consumed per day rather than servings per day. It is thus possible that inadequate fruit and vegetable consumption is overestimated in the present study, and future studies are needed to evaluate whether the number of times fruit and vegetables consumed per day corresponds to the number of servings per day. Reductions in inadequate fruit and vegetable consumption over time were only observed in three countries (Benin, United Arab Emirates, and Tonga). However, the rate of decrease was rather limited, with even the country with the most dramatic decrease (Benin) only seeing an average percent point change in prevalence per year of −1.40 (i.e., 79% in 2009 and 69.2% in 2016). In contrast, significant increases in inadequate fruit and vegetable consumption were observed in six countries (Seychelles, Jamaica, Trinidad & Tobago, Oman, Myanmar, Samoa), with the greatest average percent point change in prevalence per year in Jamaica (71.2% in 2010 and 81.2% in 2017) and Samoa (52.0% in 2011 and 60.2% in 2017). The remaining 22 countries showed a stable trend, and the prevalence of inadequate fruit and vegetable consumption was high across multiple years in most of these countries. For example, in countries such as Argentina and Maldives, the prevalence of inadequate fruit and vegetable consumption exceeded 80% across multiple years. Furthermore, the trend in most of the countries was similar for boys and girls, but the three countries observed differing trends. To the best of our knowledge, this is the first study on inadequate fruit and vegetable consumption trends, including non‐Western countries, which were predominantly LMICs, where data on this topic were non‐existent.

### 4.2 Interpretation of the Findings

Within this study, standardized methods were used across all surveys, and data showed that trends in inadequate fruit and vegetable consumption may differ substantially between countries. It is of public health concern that levels of inadequate fruit and vegetable consumption were very high in many countries and that increasing trends were more common than decreasing trends. The expansion of fast‐food options (i.e., food swamps) in such settings may also be one explanation for the increasing trends observed in some countries. For example, in Jamaica, quick‐service restaurants are the fastest growing segment of the restaurant subsector, with 50% of the market comprised of U.S. fast‐food franchises [23]. Indeed, fruit and vegetables are often lacking in U.S. fast foods. Compounding this is an increase in food insecurity observed in many settings, which is likely to reduce fruit and vegetable consumption owing to high costs [24]. Indeed, a particularly high prevalence of severe food insecurity among adolescents has been reported in countries such as Samoa, Seychelles, and Jamaica [25].

It is encouraging, however, that a significant reduction in inadequate fruit and vegetable consumption was observed in three countries. For example, in Tonga, this may be partly due to the introduction of the Tonga National Strategy to Prevent and Control Non‐Communicable Diseases 2010–2015 [26]. One key component of this strategy was healthy eating, where a plethora of actions were taken; for example, conducting healthy eating classes in schools, providing technical support for school gardening projects (growing fruit and vegetables), and enforcing policies within schools [26].

Interestingly, trends of inadequate fruit and vegetable consumption were similar between boys and girls in the majority of countries, with sex differences found in just three countries. The rationale behind the observed sex differences is unclear but could be owing to factors such as targeted campaigns, cultural norms, and preferences. For example, an increasing trend in inadequate fruit and vegetable intake was observed only among boys in Samoa. Samoa's Ministry of Health developed the Eat a Rainbow initiative, which works with schools across Samoa to teach children the benefits of eating nutritious, local produce of all the colors of the rainbow and how a varied diet promotes healthy living [27]. However, it is possible that the intervention may be more “appealing” to girls than boys, but this has yet to be evaluated. Further research of a qualitative nature is now required to elucidate on mechanisms behind such sex differences.

### 4.3 Policy Implications

Findings from the present study suggest that international campaigns are urgently required to increase fruit and vegetable consumption among adolescent boys and girls. In a recent umbrella review on interventions to increase fruit and vegetable consumption, it was found that intervention strategies implemented within schools, homes, and primary care can be effective, as can eHealth strategies, mass media campaigns, household food production strategies, and fiscal interventions [28]. When considering LMICs, major barriers to adequate fruit and vegetable consumption include socio‐demographic factors, environmental conditions, individual and cultural factors, and macrosystem influences. These barriers may be lowered at the household, school, community, and national level through multicomponent interventions, including policy and programmatic efforts to increase access to healthy and inexpensive foods (e.g., cash transfers, gardening initiatives, farm‐to‐institution programs, food baskets, nutrition–agriculture policy and program linkages, food‐market environment‐based strategies) as well as behavior change communication initiatives involving nutrition education [29]. Furthermore, the year 2021 was designated by The United Nations General Assembly as the International Year of Fruits and Vegetables (IYFV), where the Food and Agriculture Organization of the United Nations (FAO) led the implementation. The core aim of the IYFV was to increase awareness of the importance of fruit and vegetables in human nutrition, food security, and health, as well as progressing toward the United Nations Sustainable Development Goals. The IYFV steering committee developed The Global Action Plan. Included activities were developed around four main areas: (1) Advocacy and awareness raising; (2) Knowledge creation and dissemination; (3) Policy making; and (4) Capacity development and education [30].

### 4.4 Strengths and Limitations

The present work has several strengths, including the large representative sample of school‐going adolescents in 31 countries and the use of standard methodological approaches across surveys. However, when interpreting the findings, this study's limitations must be considered. First, fruit and vegetable intake was self‐reported, potentially introducing recall and social desirability bias into the findings. Future studies can utilize food diaries (including mobile technologies) and collect grocery receipts to capture more accurate fruit and vegetable consumption. Furthermore, the adequacy of fruit and vegetable consumption was based on times consumed per day rather than the number of servings per day, which is more commonly used in epidemiological studies. Second, the findings from the present study apply only to school‐going adolescents and are not generalizable to those who do not attend school, although school attendance rates are high in the majority of countries included in this study. Third, data in the present study is up to 2017, and thus, more recent trends of fruit and vegetable consumption may differ from those observed in the present work. Future research utilizing recent data is needed. Fourth, previous literature has also examined trends in fruit and vegetable consumption. However, the main limitation of the previous studies is that the data are from Western high‐income countries (with some data from Western middle‐income countries), and there were no data from other settings until now. Thus, the present study addresses this important gap in knowledge in relation to LMICs. Finally, surveys were conducted in different years depending on the country, and thus, the beta‐coefficients in our study are not totally comparable across countries. Due to this, the data should always be interpreted together with the year in which the survey was conducted.

## 5 Conclusions

This study included large representative samples of school‐going adolescents across 31 countries in Africa, Asia, and the Americas. The prevalence of inadequate fruit and vegetable intake was very high and remained so in most countries, while more countries showed significant increasing trends (*n* = 6) than decreasing trends (*n* = 3). Current global efforts to combat inadequate fruit and vegetable consumption may be insufficient, and further intensification of efforts is necessary.

## Author Contributions

**Lee Smith:** writing – original draft. **Guillermo F. López Sánchez:** writing – review and editing. **Mark A. Tully:** writing – review and editing. **Yvonne Barnett:** writing – review and editing. **Laurie Butler:** writing – review and editing. **Helen Keyes:** writing – review and editing. **Louis Jacob:** writing – review and editing. **Karel Kostev:** writing – review and editing. **Hans Oh:** writing – review and editing. **Masoud Rahmati:** writing – review and editing. **Jae Il Shin:** writing – review and editing. **Ai Koyanagi:** writing – original draft.

## Conflicts of Interest

The authors declare no conflicts of interest.

## Transparency Statement

The lead authors, Guillermo F. López Sánchez and Jae Il Shin, affirm that this manuscript is an honest, accurate, and transparent account of the study being reported; that no important aspects of the study have been omitted; and that any discrepancies from the study as planned (and, if relevant, registered) have been explained.

## Supporting information


**Table S1** Trends in prevalence (%) of inadequate fruit/vegetable consumption in 31 countries (by sex).

## Data Availability

All data is publicly available via the following link: https://www.who.int/teams/noncommunicable-diseases/surveillance/systems-tools/global-school-based-student-health-survey.
