# Supplementary material for: Temporal Trends in Inadequate Vegetable and Fruit Consumption Among Adolescents Aged 12–15 Years From 31 Countries in Asia, Africa, and the Americas
Source: Health Sci Rep. 2025 Apr 21;8(4):e70711. doi: 10.1002/hsr2.70711 (PMC12010760; doi:10.1002/hsr2.70711)
Supplement: Supplementary file 1 — Table S1 Trends in prevalence (%) of inadequate fruit/vegetable consumption in 31 countries (by sex). [file HSR2-8-e70711-s001.docx]

**APPENDIX**

| **Table S1** Trends in prevalence (%) of inadequate fruit/vegetable consumption in 31 countries (by sex) | | | | | | | | | | | |
| --- | --- | --- | --- | --- | --- | --- | --- | --- | --- | --- | --- |
|  |  | Boys | | | | | Girls | | | | |
| Country | Year | % | [95%CI] | beta^a^ | [95%CI] | P for trend^a^ | % | [95%CI] | beta^a^ | [95%CI] | P for trend^a^ |
| **AFR** |  |  |  |  |  |  |  |  |  |  |  |
| Benin | 2009 | 79.7 | [75.7,83.2] | -1.25 | [-2.26,-0.25] | 0.016 | 77.7 | [71.0,83.3] | -1.79 | [-3.22,-0.36] | 0.016 |
|  | 2016 | 70.9 | [64.9,76.3] |  |  |  | 65.2 | [57.4,72.3] |  |  |  |
| Mauritius | 2011 | 71.8 | [68.9,74.5] | -0.08 | [-0.96,0.80] | 0.857 | 74.2 | [68.1,79.5] | -0.11 | [-1.40,1.18] | 0.867 |
|  | 2017 | 71.3 | [66.9,75.3] |  |  |  | 73.6 | [68.6,78.0] |  |  |  |
| Seychelles | 2007 | 53.0 | [51.7,54.3] | 0.59 | [0.07,1.12] | 0.027 | 52.6 | [51.7,53.5] | 1.05 | [0.49,1.61] | <0.001 |
|  | 2015 | 57.8 | [53.8,61.7] |  |  |  | 61.1 | [56.6,65.3] |  |  |  |
| Swaziland | 2003 | 81.4 | [78.5,84.0] | -0.18 | [-0.70,0.33] | 0.480 | 81.5 | [79.6,83.3] | -0.21 | [-0.62,0.21] | 0.326 |
|  | 2013 | 79.5 | [74.9,83.5] |  |  |  | 79.4 | [75.5,82.9] |  |  |  |
| **AMR** |  |  |  |  |  |  |  |  |  |  |  |
| Anguilla | 2009 | 76.0 | [76.0,76.0] | 0.13 | [-0.72,0.98] | 0.762 | 79.1 | [79.1,79.1] | 0.73 | [0.08,1.38] | 0.029 |
|  | 2016 | 76.9 | [70.7,82.1] |  |  |  | 84.2 | [79.3,88.2] |  |  |  |
| Argentina | 2007 | 86.2 | [82.1,89.4] | -0.58 | [-1.39,0.23] | 0.163 | 85.2 | [80.7,88.8] | -0.68 | [-1.56,0.19] | 0.125 |
|  | 2012 | 83.3 | [81.4,85.0] |  |  |  | 81.7 | [80.0,83.3] |  |  |  |
| Guatemala | 2009 | 73.5 | [71.1,75.7] | -0.21 | [-0.90,0.48] | 0.553 | 73.4 | [68.9,77.4] | -0.59 | [-1.48,0.30] | 0.190 |
|  | 2015 | 72.2 | [68.7,75.5] |  |  |  | 69.8 | [66.6,72.9] |  |  |  |
| Guyana^b^ | 2004 | 75.6 | [70.8,79.8] | -1.62 | [-2.71,-0.53] | 0.005 | 72.2 | [66.3,77.4] | -0.25 | [-1.68,1.18] | 0.725 |
|  | 2010 | 65.8 | [61.3,70.1] |  |  |  | 70.7 | [64.2,76.4] |  |  |  |
| Jamaica | 2010 | 69.9 | [65.1,74.3] | 1.06 | [0.03,2.08] | 0.043 | 73.4 | [68.9,77.4] | 1.65 | [0.78,2.51] | <0.001 |
|  | 2017 | 77.3 | [71.7,82.1] |  |  |  | 84.9 | [80.5,88.5] |  |  |  |
| Suriname | 2009 | 67.2 | [60.9,72.9] | 0.47 | [-0.66,1.61] | 0.399 | 70.6 | [66.3,74.7] | 0.11 | [-0.65,0.87] | 0.770 |
|  | 2016 | 70.5 | [65.6,74.9] |  |  |  | 71.4 | [68.5,74.2] |  |  |  |
| Trinidad & Tobago | 2007 | 75.3 | [70.2,79.7] | 0.26 | [-0.33,0.84] | 0.386 | 76.1 | [72.9,79.0] | 0.74 | [0.37,1.11] | <0.001 |
|  | 2011 | 83.7 | [79.7,87.1] |  |  |  | 83.5 | [79.8,86.6] |  |  |  |
|  | 2017 | 78.4 | [74.7,81.8] |  |  |  | 83.9 | [81.6,86.0] |  |  |  |
| Uruguay | 2006 | 77.2 | [74.3,79.9] | -0.57 | [-1.19,0.06] | 0.078 | 76.8 | [73.6,79.7] | 0.16 | [-0.55,0.88] | 0.648 |
|  | 2012 | 73.8 | [71.3,76.2] |  |  |  | 77.8 | [74.7,80.6] |  |  |  |
| **EMR** |  |  |  |  |  |  |  |  |  |  |  |
| Egypt | 2006 | 77.6 | [71.1,83.0] | -0.64 | [-2.76,1.48] | 0.549 | 80.7 | [74.8,85.5] | -1.05 | [-2.79,0.70] | 0.234 |
|  | 2011 | 74.4 | [65.1,81.9] |  |  |  | 75.5 | [68.2,81.5] |  |  |  |
| Jordan | 2004 | 76.7 | [73.1,80.0] | -1.57 | [-3.25,0.12] | 0.067 | 73.8 | [67.9,79.0] | 0.84 | [-1.92,3.61] | 0.538 |
|  | 2007 | 72.0 | [68.6,75.2] |  |  |  | 76.4 | [70.3,81.5] |  |  |  |
| Kuwait | 2011 | 74.5 | [69.9,78.5] | 1.63 | [0.12,3.13] | 0.035 | 81.3 | [77.2,84.8] | 0.03 | [-1.23,1.29] | 0.959 |
|  | 2015 | 81.0 | [76.9,84.5] |  |  |  | 81.4 | [78.4,84.1] |  |  |  |
| Lebanon | 2005 | 72.0 | [69.7,74.2] | 0.06 | [-0.31,0.43] | 0.761 | 77.8 | [75.9,79.7] | 0.06 | [-0.19,0.31] | 0.652 |
|  | 2011 | 68.3 | [64.7,71.7] |  |  |  | 75.9 | [72.9,78.6] |  |  |  |
|  | 2017 | 72.5 | [68.6,76.0] |  |  |  | 78.4 | [76.1,80.5] |  |  |  |
| Morocco | 2006 | 63.4 | [59.6,66.9] | 0.19 | [-0.39,0.76] | 0.515 | 62.2 | [58.9,65.3] | 0.55 | [-0.01,1.11] | 0.053 |
|  | 2010 | 56.8 | [52.9,60.7] |  |  |  | 49.1 | [46.3,52.0] |  |  |  |
|  | 2016 | 63.7 | [59.3,68.0] |  |  |  | 64.4 | [60.2,68.4] |  |  |  |
| Oman | 2005 | 66.0 | [62.4,69.4] | 0.81 | [0.23,1.39] | 0.007 | 72.8 | [70.3,75.2] | 0.78 | [0.34,1.22] | 0.001 |
|  | 2010 | 72.0 | [64.5,78.4] |  |  |  | 78.1 | [74.2,81.5] |  |  |  |
|  | 2015 | 73.7 | [69.0,78.0] |  |  |  | 80.4 | [76.5,83.9] |  |  |  |
| United Arab Emirates | 2005 | 78.7 | [77.2,80.1] | -0.50 | [-0.83,-0.17] | 0.003 | 83.2 | [82.0,84.4] | -0.44 | [-0.84,-0.04] | 0.030 |
|  | 2010 | 78.9 | [75.4,82.0] |  |  |  | 84.3 | [81.8,86.5] |  |  |  |
|  | 2016 | 73.4 | [70.1,76.5] |  |  |  | 78.7 | [74.4,82.4] |  |  |  |
| Yemen | 2008 | 83.4 | [78.5,87.3] | -0.24 | [-1.46,0.98] | 0.691 | 87.8 | [74.7,94.6] | -2.20 | [-4.30,-0.10] | 0.041 |
|  | 2014 | 81.9 | [75.8,86.8] |  |  |  | 74.6 | [66.5,81.3] |  |  |  |
| **SEAR** |  |  |  |  |  |  |  |  |  |  |  |
| Indonesia | 2007 | 74.6 | [71.5,77.4] | -0.03 | [-0.51,0.45] | 0.903 | 76.0 | [72.1,79.4] | 0.02 | [-0.51,0.55] | 0.940 |
|  | 2015 | 74.4 | [72.0,76.6] |  |  |  | 76.1 | [74.1,78.0] |  |  |  |
| Maldives | 2009 | 84.2 | [79.6,88.0] | 0.53 | [-0.44,1.50] | 0.285 | 90.8 | [87.8,93.1] | 0.46 | [-0.22,1.14] | 0.182 |
|  | 2014 | 86.9 | [84.3,89.1] |  |  |  | 93.1 | [90.6,94.9] |  |  |  |
| Myanmar | 2007 | 83.4 | [80.0,86.3] | 0.75 | [0.33,1.16] | 0.001 | 83.7 | [78.7,87.7] | 0.63 | [0.04,1.22] | 0.038 |
|  | 2016 | 90.1 | [88.2,91.7] |  |  |  | 89.3 | [86.3,91.7] |  |  |  |
| Sri Lanka | 2008 | 79.2 | [75.3,82.7] | -0.25 | [-0.91,0.41] | 0.445 | 75.2 | [71.0,78.9] | -0.09 | [-0.87,0.69] | 0.818 |
|  | 2016 | 77.2 | [73.5,80.5] |  |  |  | 74.5 | [69.7,78.7] |  |  |  |
| Thailand | 2008 | 64.9 | [60.8,68.8] | 0.63 | [-0.07,1.34] | 0.079 | 66.9 | [62.0,71.5] | 0.54 | [-0.37,1.45] | 0.236 |
|  | 2015 | 69.3 | [66.5,72.0] |  |  |  | 70.7 | [66.5,74.6] |  |  |  |
| **WPR** |  |  |  |  |  |  |  |  |  |  |  |
| Cooks Island | 2011 | 58.2 | [58.2,58.2] | 0.58 | [-1.19,2.34] | 0.513 | 61.5 | [61.5,61.5] | 0.44 | [-0.95,1.82] | 0.527 |
|  | 2015 | 60.5 | [53.5,67.1] |  |  |  | 63.2 | [57.7,68.4] |  |  |  |
| Fiji | 2010 | 60.1 | [53.8,66.0] | 0.62 | [-0.82,2.06] | 0.387 | 62.4 | [55.6,68.7] | -0.16 | [-1.81,1.49] | 0.842 |
|  | 2016 | 63.8 | [58.0,69.1] |  |  |  | 61.4 | [54.4,68.0] |  |  |  |
| Philippines | 2003 | 73.9 | [70.3,77.1] | -0.11 | [-0.43,0.22] | 0.517 | 76.7 | [73.5,79.6] | -0.28 | [-0.60,0.04] | 0.087 |
|  | 2007 | 77.0 | [74.5,79.3] |  |  |  | 79.7 | [77.5,81.7] |  |  |  |
|  | 2011 | 73.5 | [69.1,77.5] |  |  |  | 75.6 | [72.4,78.5] |  |  |  |
|  | 2015 | 74.0 | [71.5,76.3] |  |  |  | 74.7 | [72.0,77.3] |  |  |  |
| Samoa^b^ | 2011 | 50.8 | [45.5,56.2] | 2.15 | [0.91,3.38] | 0.001 | 53.1 | [49.3,56.9] | 0.65 | [-0.21,1.51] | 0.136 |
|  | 2017 | 63.7 | [58.7,68.4] |  |  |  | 57.0 | [53.7,60.3] |  |  |  |
| Tonga | 2010 | 62.4 | [57.7,67.0] | -1.05 | [-1.85,-0.25] | 0.010 | 59.4 | [55.5,63.1] | -0.78 | [-1.53,-0.04] | 0.039 |
|  | 2017 | 55.1 | [52.1,58.0] |  |  |  | 53.9 | [50.4,57.4] |  |  |  |
| Vanuatu^b^ | 2011 | 51.7 | [45.3,58.1] | -1.71 | [-3.49,0.07] | 0.059 | 45.5 | [39.9,51.2] | 0.55 | [-1.10,2.20] | 0.506 |
|  | 2016 | 43.2 | [37.5,49.1] |  |  |  | 48.2 | [42.5,54.0] |  |  |  |

Abbreviation: CI Confidence interval; AFR African Region; AMR Region of the Americas; EMR Eastern Mediterranean Region; SEAR South-East Asia Region; WPR Western Pacific Region

^a^ The beta and P for trend are based on linear regression including survey year as a continuous variable. The beta can be interpreted as the average percentage point change in prevalence per year.

^b^ Significant interaction in trends by sex (P<0.05).
